# Fostering sustainable infection prevention behaviors through organizational culture and psychological resources in healthcare settings

**DOI:** 10.1097/MD.0000000000045013

**Published:** 2025-10-03

**Authors:** Sun Ok Kim, Moon Suk Sim

**Affiliations:** a Department of Nursing, Sunlin University, Pohang-si, Gyeongsangbuk-do, Republic of Korea; b Department of Nursing, Konyang University, Daejeon, Republic of Korea.

**Keywords:** attitudes, infection prevention, job stress, knowledge, organizational culture, self-efficacy

## Abstract

Infection prevention is a critical aspect of healthcare, directly impacting patient safety and outcomes. Previous studies have investigated various individual factors influencing infection control behaviors; however, limited research has examined the combined impact of organizational culture, environmental factors, and self-efficacy. This study aims to explore the multidimensional determinants of infection prevention behaviors using the information–motivation–behavioral skills framework. A cross-sectional study was conducted among 407 nurses from 2 mid-sized hospitals in South Korea. Data were collected using a structured survey assessing infection control organizational culture, prevention environment, knowledge, attitudes, job stress, self-efficacy, and prevention behaviors. Structural equation modeling was applied to analyze direct and indirect relationships between variables. Infection control organizational culture significantly influenced both infection control attitudes and prevention behaviors, while the prevention environment had a significant effect on job stress. Infection control self-efficacy emerged as a critical mediator, significantly enhancing prevention behaviors. Contrary to expectations, job stress did not directly undermine infection prevention behaviors but indirectly influenced them through self-efficacy. Knowledge had limited direct effects but influenced prevention behaviors through attitudes and self-efficacy. The findings underscore the importance of fostering a supportive organizational culture and prevention environment to enhance infection control practices. By integrating the Information–Motivation–Behavioral Skills Model framework, this study highlights the interconnected pathways influencing infection prevention behaviors. These insights can guide the development of targeted interventions and policies to improve healthcare safety and outcomes.

## 1. Introduction

Infection prevention and control (IPC) practices in healthcare settings are vital for safeguarding patient safety and reducing healthcare-associated infections (HAIs).^[1]^ Globally, HAIs contribute significantly to patient morbidity, prolonged hospital stays, and increased healthcare costs.^[2]^ The importance of IPC has been underscored by guidelines from institutions such as the Centers for Disease Control and Prevention, which emphasize evidence-based practices including hand hygiene, personal protective equipment (PPE) use, and environmental cleaning.^[3]^ Research has consistently shown that effective IPC measures are influenced by multiple factors, including healthcare professionals’ knowledge, attitudes, organizational culture, and environmental support.^[4,5]^ The integration of behavioral models, such as the Information–Motivation–Behavioral Skills (IMB) Model, has further enhanced understanding of the behavioral dynamics underlying compliance with IPC protocols.^[6]^ These studies highlight the significance of personal and contextual factors in driving infection prevention behaviors in clinical settings.

Despite substantial progress in understanding IPC determinants, gaps remain in the literature. First, existing research often isolates specific factors, such as knowledge or organizational culture, without fully exploring their interrelationships within a broader theoretical framework.^[7–10]^ Second, the role of healthcare professionals’ psychological experiences, such as job stress and self-efficacy, in influencing IPC compliance has received limited attention. While stress is known to undermine cognitive and emotional functioning, its impact on infection prevention behaviors through self-efficacy and attitudes remains underexplored.^[11,12]^ Furthermore, the dynamics of how organizational and environmental factors shape these psychological and behavioral pathways are insufficiently investigated in diverse healthcare settings, particularly mid-sized hospitals. Finally, most studies focus on general compliance rates, overlooking the nuanced mechanisms by which IPC behaviors are fostered and sustained.

Addressing these gaps is essential given the profound implications of IPC for public health and patient safety. The ongoing COVID-19 pandemic has magnified the importance of robust IPC measures, particularly in mitigating the spread of infectious diseases in healthcare environments.^[13]^ Understanding the multidimensional determinants of IPC behaviors can inform more effective interventions, addressing not only individual knowledge and attitudes but also broader organizational and environmental contexts. Failure to investigate these complex interdependencies risks perpetuating preventable HAIs, placing undue strain on healthcare systems and jeopardizing patient outcomes.^[14]^ Moreover, exploring the mediating roles of psychological constructs such as self-efficacy can offer actionable insights into how healthcare workers can be better supported to overcome barriers to IPC compliance.

This study aims to address these gaps by applying the IMB model, extended with additional variables, to investigate the determinants of infection prevention behaviors among healthcare professionals. Specifically, the study examines how organizational culture, prevention environment, knowledge, attitudes, job stress, and self-efficacy interact to influence IPC practices. Organizational culture is explored as a driver of shared norms and values, fostering consistent IPC behaviors.^[15]^ The prevention environment is conceptualized as the availability of resources and administrative support that enable compliance.^[5]^ Psychological constructs such as attitudes, job stress, and self-efficacy are examined as mediators that link contextual factors to behavioral outcomes. Using structural equation modeling, this study seeks to elucidate direct and indirect relationships, providing a comprehensive view of the pathways shaping IPC compliance in healthcare settings.

This study contributes to the literature in several significant ways. First, it offers an integrated framework for understanding IPC behaviors, combining organizational, environmental, and psychological factors within the IMB model. By identifying the interplay between these determinants, the study advances theoretical insights into IPC compliance, addressing gaps in previous research that often isolated these factors.^[4,6]^ Second, the study highlights the mediating role of self-efficacy, demonstrating its critical importance in translating knowledge, attitudes, and environmental support into consistent behaviors. This finding aligns with and extends existing research on self-efficacy’s role in healthcare settings.^[16,17]^ Third, the study provides actionable insights for practitioners, including healthcare managers and policy-makers, by identifying organizational and environmental levers that can enhance IPC compliance. The results underscore the importance of fostering a positive organizational culture and providing adequate resources to reduce job stress and enhance self-efficacy. By addressing these elements, healthcare systems can develop more targeted and effective interventions to improve patient safety and reduce HAIs.

In summary, this study addresses critical gaps in the IPC literature, offering a nuanced understanding of the multidimensional factors influencing infection prevention behaviors in healthcare settings. The findings not only advance theoretical understanding but also offer practical guidance for improving IPC compliance, contributing to safer and more efficient healthcare systems.

## 2. Theoretical framework

**Figure 1.**
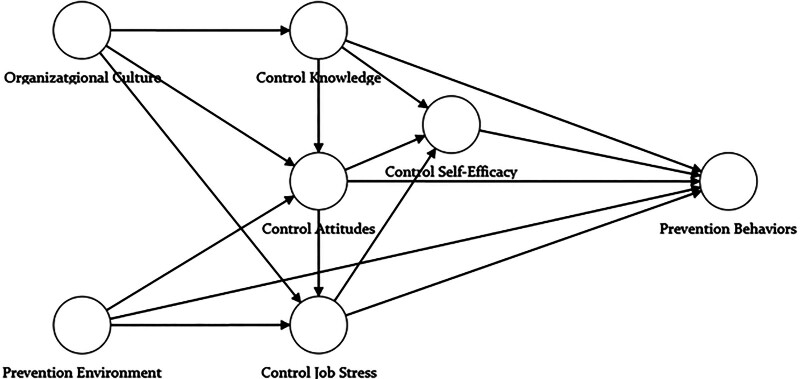
Research framework.

The theoretical framework of this study is based on the IMB model proposed by Fisher and Fisher,^[6]^ a widely recognized framework for understanding health-related behaviors. The IMB model posits that information, motivation, and behavioral skills are essential determinants of successful behavior change. Information refers to accurate knowledge relevant to the target behavior, while motivation encompasses personal and social incentives to engage in the behavior. Behavioral skills involve the ability to translate information and motivation into action. To address the complexity of infection control behaviors in healthcare, this study extends the IMB model by incorporating additional variables such as infection control organizational culture, prevention environment, and job stress. These extensions aim to better capture the multifaceted influences on healthcare professionals’ infection prevention behaviors. Figure 1 illustrates the research framework.

### 2.1. Infection control organizational culture

Infection control organizational culture refers to shared norms, values, and practices within a healthcare setting that prioritize infection prevention and control measures.^[18]^ Such culture fosters an environment where infection control behaviors are not only expected but systematically supported through policies, communication, and leadership commitment. Research highlights that an organization’s culture significantly influences how healthcare professionals perceive and engage with infection prevention protocols.^[4,19]^ For example, collaborative and transparent practices within an organization can empower healthcare workers to prioritize infection control behaviors.^[10,12]^ Strong organizational cultures also enhance individual attitudes toward compliance with infection prevention measures by reducing perceived barriers and promoting proactive engagement.^[15]^ The importance of an infection control-focused culture is further underscored by evidence suggesting that it supports ongoing education and awareness, which are vital for sustaining infection prevention efforts in clinical settings.^[3]^ Building on these findings, this study proposes that infection control organizational culture positively influences infection control knowledge, attitudes, and behaviors among healthcare professionals.

H1a: Infection control organizational culture influences infection control knowledge.

H1b: Infection control organizational culture influences infection control attitudes.

H1c: Infection control organizational culture influences the performance of infection prevention behaviors.

### 2.2. Infection control prevention environment

Infection control prevention environment refers to the physical, administrative, and resource-based infrastructure designed to facilitate infection prevention efforts in healthcare settings.^[5]^ This includes the availability of PPE, adequate training, standardized protocols, and administrative support systems that ensure adherence to infection control measures.^[20,21]^ The presence of a supportive prevention environment is crucial for creating a safe and efficient workplace, enabling healthcare professionals to adopt positive attitudes toward infection prevention.^[22,23]^ Moreover, research suggests that well-resourced and organized prevention environments can alleviate occupational stress by reducing uncertainties and barriers associated with implementing infection control measures.^[24]^ Such environments also enhance compliance by minimizing logistical challenges and promoting ease of access to necessary tools and training. These findings underscore the importance of fostering robust prevention environments to ensure that infection control measures are effectively implemented and sustained. Based on these insights, this study posits that infection control prevention environments influence attitudes, job stress, and the performance of infection prevention behaviors in healthcare settings.

H2a: Infection control prevention environment influences infection control attitudes.

H2b: Infection control prevention environment influences infection control job stress.

H3c: Infection control prevention environment influences the performance of infection prevention behaviors.

### 2.3. Infection control knowledge

Infection control knowledge encompasses healthcare professionals’ understanding of evidence-based practices, guidelines, and protocols essential for preventing and controlling infections in clinical settings.^[25]^ Knowledge serves as a foundation for informed decision-making, equipping individuals with the capability to recognize risks and implement appropriate preventive measures.^[26]^ Studies have shown that comprehensive infection control knowledge significantly shapes healthcare workers’ perceptions and attitudes, fostering a proactive approach to infection prevention.^[7,27]^ Additionally, knowledge has been identified as a critical determinant of self-efficacy, as it instills confidence in one’s ability to perform infection control tasks effectively and handle challenges encountered during implementation.^[28]^ Evidence further suggests that increased infection control knowledge enhances behavioral compliance by promoting adherence to best practices and reinforcing the importance of consistent application of preventive measures.^[29]^ Drawing from these findings, this study proposes that infection control knowledge influences attitudes, self-efficacy, and the performance of infection prevention behaviors among healthcare professionals.

H3a: Infection control knowledge influences infection control attitudes.

H3b: Infection control knowledge influences infection control self-efficacy.

H3c: Infection control knowledge influences the performance of infection prevention behaviors.

### 2.4. Infection control attitude

Infection control attitude refers to healthcare professionals’ mental disposition, beliefs, and emotional responses toward infection prevention and control practices.^[30]^ Attitudes play a pivotal role in determining how individuals perceive and respond to infection-related challenges, shaping their motivation to engage with preventative behaviors.^[31]^ Positive attitudes have been shown to mitigate job-related stress by fostering a sense of purpose and control in addressing infection risks, thus alleviating emotional burdens.^[14]^ Moreover, research suggests that constructive attitudes significantly enhance self-efficacy by reinforcing confidence in one’s ability to effectively perform infection prevention tasks, even in demanding circumstances.^[16,32]^ Strong infection control attitudes also drive consistent behavioral compliance, as they create an intrinsic motivation to adhere to infection prevention protocols, regardless of external enforcement.^[33]^ Building on this evidence, this study posits that infection control attitudes influence job stress, self-efficacy, and the execution of infection prevention behaviors among healthcare professionals.

H4a: Infection control attitudes influences infection control job stress.

H4b: Infection control attitudes influences infection control self-efficacy.

H4c: Infection control attitudes influences the performance of infection prevention behaviors.

### 2.5. Infection control job stress

Infection control job stress refers to the psychological strain experienced by healthcare workers when managing infection prevention responsibilities, including workload, resource limitations, and time constraints.^[34]^ High job stress can impair cognitive and emotional functioning, reducing confidence in handling infection control tasks and affecting performance consistency.^[35]^ Research suggests that excessive stress undermines self-efficacy by fostering feelings of inadequacy and diminishing problem-solving capacity.^[11,36]^ Moreover, stress negatively impacts compliance with infection prevention behaviors, as overwhelmed individuals may deprioritize adherence to protocols due to fatigue or time pressures.^[12,35]^ Conversely, stress reduction has been linked to improved focus, decision-making, and adherence to infection control measures.^[13]^ Based on these findings, this study explores the relationship between infection control job stress, self-efficacy, and the execution of infection prevention behaviors.

H5a: Infection control job stress influences infection control self-efficacy.

H5b: Infection control job stress influences the performance of infection prevention behaviors.

### 2.6. Infection control self-efficacy

Infection control self-efficacy refers to healthcare workers’ confidence in their ability to effectively perform infection prevention measures.^[28]^ High self-efficacy is associated with increased motivation and persistence, enabling individuals to overcome barriers and consistently adhere to infection control protocols.^[16]^ Studies demonstrate that self-efficacy enhances compliance with preventive behaviors, particularly in high-pressure situations, by reinforcing confidence in one’s skills and decision-making.^[37,38]^ It also plays a critical role in fostering proactive engagement with infection prevention practices, improving behavioral consistency.^[17]^ This study proposes that self-efficacy positively influences infection prevention behaviors in clinical settings.

H6: Infection control self-efficacy influences the performance of infection prevention behaviors.

## 3. Methods

### 3.1. Ethical consideration

This study was conducted after receiving approval from the Institutional Review Board of Konyang University (IRB File No. KYU 2023-05-027-001).

### 3.2. Measure development

A structured questionnaire comprising 153 items was utilized, and permission to use all instruments was obtained from the original authors via email.

#### 3.2.1. Infection control organizational culture

The measurement tool was adapted from the organizational culture instrument developed by the Agency for Healthcare Research and Quality, translated by Kim et al,^[39]^ modified for nurses by Park,^[40]^ and further refined by Moon and Song^[41]^ for infection management guidelines. The tool consisted of 10 items rated on a 7-point Likert scale (1 = strongly disagree, 7 = strongly agree), with scores ranging from 10 to 70. Higher scores indicated a more positive perception of infection control organizational culture. The reliability in this study was Cronbach α = .92.

#### 3.2.2. Infection control prevention environment

This variable was measured using the defense environment instrument developed by Han,^[42]^ modified for emergency nurses by Ahn et al.^[43]^ The tool consisted of 11 items rated on a 5-point Likert scale (1 = never true, 5 = always true), with scores ranging from 11 to 55. Higher scores reflected a better infection control prevention environment. The reliability in this study was Cronbach α = .91.

#### 3.2.3. Infection control knowledge

The tool developed by Cho and Choi,^[44]^ later revised by Suh and Oh^[45]^ and Baek^[46]^ based on the updated Centers for Disease Control and Prevention standard precautions guidelines, was used. Responses included “yes,” “no,” and “do not know,” with correct answers scored as 1 and others as 0. Scores ranged from 0 to 29, with higher scores indicating greater infection control knowledge. The reliability was Kuder–Richardson 20 = .52.

#### 3.2.4. Infection control attitudes

This variable was measured using a tool revised by Kim,^[47]^ consisting of 12 items rated on a 5-point Likert scale (1 = strongly disagree, 5 = strongly agree). Two reverse-scored items were included. Scores ranged from 11 to 60, with higher scores indicating more positive attitudes. The reliability was Cronbach α = .84.

#### 3.2.5. Infection control job stress

Huh^[48]^ tool, revised for emergency settings by Jang,^[49]^ was used. It included 32 items rated on a 5-point Likert scale (1 = no stress, 5 = extreme stress), with scores ranging from 32 to 160. Higher scores reflected greater job stress. The reliability was Cronbach α = .98.

#### 3.2.6. Infection control self-efficacy

This study adapted Sherer^[50]^ tool, comprising 22 items rated on a 5-point Likert scale (1 = strongly disagree, 5 = strongly agree). Scores ranged from 22 to 110, with higher scores indicating higher self-efficacy. The reliability in this study was Cronbach α = .98.

#### 3.2.7. Infection prevention behaviors

The tool developed by Lee et al^[51]^ based on the Korean Disease Control and Prevention Agency COVID-19 response guidelines and revised by Park^[52]^ was used. It consisted of 18 items rated on a 4-point Likert scale (1 = never performed, 4 = always performed), with scores ranging from 18 to 72. Higher scores reflected better infection prevention performance. Reliability was Cronbach α = .95.

### 3.3. Settings and participants

The survey was conducted from June 23 to July 20, 2023, targeting nurses working in 2 mid-sized hospitals (N Hospital and B Hospital) in Incheon, South Korea. A total of 407 responses were included in the final analysis. Participants were selected based on voluntary consent, communication ability, and employment in hospitals with 100 to 300 beds for at least 3 months.

### 3.4. Data collection

Prior to the survey, the researcher explained the study’s purpose to the nursing department heads and obtained approval following each institution’s protocol. Data collection began after confirming participants’ consent and eligibility. Surveys were administered until the target sample size of 407 responses was reached, with all responses deemed complete and used for the final analysis.

## 4. Results

### 4.1. Sample characteristics

The sample demographics of this study, summarized in Table 1, include 407 participants, with 12% male and 88% female nurses. The average age was 45.5 years. In terms of education, 26.8% held associate degrees, 70.5% had bachelor’s degrees, and 2.7% held master’s degrees or higher. Regarding religion, 27.3% reported having a religious affiliation, while 72.7% did not. A majority were single (58.5%), while 41.5% were married. Total work experience varied, with 27.8% having 1 to 4 years and 22.4% having 10 to 20 years. The largest group for current experience was 1 to 4 years (45.7%). Most participants worked in general wards (48.2%), followed by 13% in emergency rooms. Over half worked 3 shifts (57.7%), and 75.2% were general nurses. Training needs were reported by 82.3%, and 86% had received training. Key infection control challenges cited included lack of time (43.2%) and overwork (30.2%).

**Table 1:** Sample demographics.

Characteristic	Category	Frequency (n)	Percentage (%)
Gender	Male	49	12
	Female	358	88
Age	22–29	185	45.5
	30–39	120	29.5
	40–49	76	18.6
	50–59	26	6.4
Education	Associate degree	109	26.8
	Bachelor’s	287	70.5
	Master’s or higher	11	2.7
Religion	Yes	111	27.3
	No	296	72.7
Marital Status	Single	238	58.5
	Married	169	41.5
Total Experience	<1 yr	49	12
	1–4 yr	113	27.8
	4–7 yr	57	14
	7–10 yr	51	12.5
	10–20 yr	91	22.4
	20+ yr	46	11.3
Current experience	<1 yr	124	30.5
	1–4 yr	186	45.7
	7–10 yr	33	8.1
	10–20 yr	57	14
	20 yr	7	1.7
Department	General ward	196	48.2
	ICU	29	7.1
	ER	53	13
	Outpatient	36	8.8
	Other	93	22.9
Position	General nurse	306	75.2
	Senior nurse	28	6.9
	Charge nurse	31	7.6
	Head nurse or above	42	10.3
Work type	Three shifts	235	57.7
	Two shifts	21	5.2
	Daytime	123	30.2
	Other	28	6.9
Need for training	Yes	335	82.3
	No	72	17.7
Received training	Yes	350	86
	No	57	14
Number of trainings	None	57	14
	Once	171	42
	Twice	92	22.6
	Three times	41	10.1
	Four or more times	46	11.3
	None	65	16
PPE training	Once	165	40.5
	Twice	91	22.4
	Three times	37	9.1
	Four or more times	49	12
	Lack of time	176	43.2
Reasons for not performing infection control	Lack of facilities	31	7.6
	Lack of knowledge	18	4.5
	Overwork	123	30.2
	Inconvenience	26	6.4
	Other	33	8.1
	Yes	334	82.1
Nursing Role	No	73	17.9
	Hand hygiene	364	89.5
Important Area	Fluid therapy	3	0.7
	Urinary tract infection control	2	0.5
	Respiratory infection control	27	6.6
	Medical waste management	4	1
	Disinfection and contaminated equipment	7	1.7
Infection control performed	Yes	334	82.1
	No	73	17.9
Isolation room availability	Yes	381	93.6
	No	26	6.4

ER = emergency room, ICU = intensive care unit, PPE = personal protective equipment.

### 4.2. Measurement model

The measurement model was evaluated for reliability, validity, and discriminant validity, as shown in Tables 2 and 3. Reliability was assessed using construct reliability (CR) and item factor loadings. All constructs demonstrated high reliability, with CR values exceeding the recommended threshold of 0.7.^[53]^ For example, infection control organizational culture showed a CR of.92, and infection control self-efficacy demonstrated the highest CR of.99 (Table 2).

**Table 2:** Reliability and validity.

Construct	Item	URW	SRW	SE	C.R.	*P*	CR	AVE
Infection control organizational culture	Communication	1	0.923				.92	.81
	Culture	0.943	0.913	0.032	29.396	<.001		
	Manager attitude	0.995	0.856	0.039	25.522	<.001		
Infection control prevention environment	Equipment administrative environment	1	0.555				.90	.65
		1.805	0.972	0.151	11.922	<.001		
	Facility environment							
	Standard precautions	1.514	0.843	0.126	12.027	<.001		
Infection control knowledge	Hand hygiene	1	0.653				.67	.41
	PPE	0.595	0.517	0.112	5.316	<.001		
	Communication	0.963	0.507	0.181	5.314	<.001		
Infection control attitudes		0.397	0.919					.84
Infection control job stress	Quantitative workload	1	0.826				.95	.79
	Qualitative workload	1.062	0.926	0.044	24.13	<.001		
	Interpersonal conflicts	1.135	0.901	0.049	23.086	<.001		
	Organizational factors	1.186	0.904	0.051	23.208	<.001		
Infection control self-efficacy	Confidence	1	0.919				.99	.90
	Achievement	1.001	0.947	0.028	36.357	<.001		
	Behavior initiation	1.009	0.961	0.026	38.555	<.001		
	Effort	1.007	0.968	0.025	39.69	<.001		
	Persistence	1.044	0.953	0.028	37.272	<.001		
Infection prevention behavior performance	Hand hygiene performance	1	0.748				.96	.66
	PPE management performance	1.049	0.841	0.06	17.519	<.001		
	Disinfection and contamination control	1.261	0.87	0.069	18.208	<.001		
	Isolation practice	1.033	0.862	0.057	18.021	<.001		
	Medical waste management	1.338	0.815	0.079	16.902	<.001		
	Health management	1.274	0.734	0.085	15.052	<.001		

AVE = average variance extracted, C.R. = critical ratio, CR = construct reliability, PPE = personal protective equipment, SE = standard error, SRW = standardized regression weight, URW = unstandardized regression weight.

**Table 3:** Correlation and discriminant validity.

Construct	1	2	3	4	5	6	7
1. Infection control organizational culture	.81						
2. Infection control prevention environment	.467	.65					
	.218						
3. Infection control knowledge	−.063	.040	.41				
	.004	.002					
4. Infection control attitudes	.560	.307	.032	.84			
	.314	.001	.001				
5. Infection control job stress	−.142	−.164	−.007	−.005	.79		
	.020	.027	<.001	<.001			
6. Infection control self-efficacy	.469	.388	.098	.533	−.025	.90	
	.220	.151	.010	.306	.001		
7. Infection prevention behavior performance	.507	.332	−.085	.451	−.029	.536	.66
	.257	.110	.007	.203	.001	.287	

The diagonal matrix values represent the AVE of each factor.

AVE = average variance extracted.

Validity was assessed through convergent and discriminant validity. Convergent validity was confirmed as the average variance extracted values for all constructs exceeded the 0.5 threshold, such as.81 for infection control organizational culture and.66 for infection prevention behavior performance.

Discriminant validity was supported as the square root of average variance extracted for each construct exceeded the inter-construct correlations, for instance, .81 for infection control organizational culture compared to its highest correlation of .560 with infection control attitudes (Table 3).

These results indicate that the measurement model meets the required standards of reliability and validity.

### 4.3. Model fit

The model fit was assessed using multiple fit indices to evaluate the adequacy of the hypothesized model, as shown in Table 4. The Chi-square (*χ*²) value was significant at 617.531 (*P* < .001), indicating a lack of absolute fit; however, Chi-square is sensitive to sample size and is often complemented by other indices.^[54]^ The Chi-square minimum discrepancy per degree of freedom ratio was 2.384, falling below the threshold of 3, which demonstrates an excellent fit.^[53]^

**Table 4:** Model fit.

Fit index	Criterion	Hypothetical model
*χ*^2^ (CMIN)	*P* ≥ .05 acceptable	617.531 (*P* < .001)
DF	–	259
CMIN/DF	≤3 excellent	2.384
GFI	≥.9 excellent, ≥.8 acceptable	.891
AGFI	≥.85 excellent, ≥.8 acceptable	.864
NFI	≥.9 excellent	.933
TLI	≥.9 excellent	.953
CFI	≥.9 excellent	.960
RMSEA	≤.06 excellent, ≤.08 acceptable	.058
SRMR	≤.08 excellent	.051

AGFI = Adjusted Goodness of Fit Index, CFI = Comparative Fit Index, CMIN/DF = Chi-square minimum discrepancy per degree of freedom, DF = degrees of freedom, GFI = Goodness of Fit Index, NFI = Normed Fit Index, RMSEA = root mean squared error of approximation, SRMR = standardized root mean residual, TLI = Tucker–Lewis Index.

Incremental fit indices such as Goodness of Fit Index (.891), Adjusted Goodness of Fit Index (.864), Normed Fit Index (.933), Tucker–Lewis Index (.953), and Comparative Fit Index (.960) exceeded or approached the recommended cutoff values for acceptable or excellent fit. Additionally, error-based indices including root mean square error of approximation (.058) and standardized root mean residual (.051) were within the acceptable thresholds, indicating strong model fit. These results suggest that the hypothesized model provides an adequate representation of the observed data.

### 4.4. Structural model

The structural equation modeling (SEM) results highlight the relationships between the constructs in the proposed model. infection control organizational culture significantly influenced infection control attitudes (β = 0.738, *P* < .001), while its effect on infection control knowledge was not significant (β = −0.064, *P* = .340). infection control prevention environment significantly reduced infection control job stress (β = −0.163, *P* = .007) but did not have a significant effect on infection control attitudes (β = 0.096, *P* = .084). infection control self-efficacy was strongly impacted by infection control attitudes (β = 0.637, *P* < .001), whereas infection prevention behavior performance was positively associated with both infection control organizational culture (β = 0.207, *P* = .047) and self-efficacy (β = 0.335, *P* < .001). These findings underscore the critical roles of organizational culture and self-efficacy in driving infection prevention behaviors. Further details are presented in Table 5.

**Table 5:** Summary of SEM results.

H	Exogenous (Cause variable)	Endogenous (result variable)	RW (B)	SRW (β)	SE	C.R. (*t* value)	*P*	SMC
H1a	Organizational culture	Control knowledge	−0.036	−0.064	0.038	−0.955	.340	0.004
H1b	Organizational culture	Control attitudes	0.284	0.738	0.025	11.463	.000	0.619
H1c	Organizational culture	Prevention behaviors	0.103	0.207	0.052	1.985	.047	0.390
H2a	Prevention environment	Control attitudes	0.052	0.096	0.030	1.729	.084	0.619
H2b	Prevention environment	Control job stress	−0.222	−0.163	0.082	−2.704	.007	0.027
H2c	Prevention environment	Prevention behaviors	0.033	0.047	0.036	0.922	.356	0.390
H3a	Knowledge	Control attitudes	0.054	0.079	0.047	1.148	.251	0.619
H3b	Knowledge	Control self-efficacy	0.091	0.076	0.074	1.231	.218	0.413
H3c	Knowledge	Prevention behaviors	−0.097	−0.111	0.050	−1.921	.055	0.390
H4a	Attitudes	Control job stress	−0.010	−0.004	0.171	−0.056	.955	0.027
H4b	Attitudes	Control self-efficacy	1.136	0.637	0.113	10.063	.000	0.413
H4c	Attitudes	Prevention behaviors	0.194	0.150	0.187	1.038	.299	0.390
H5a	Stress	Control self-efficacy	0.016	0.022	0.032	0.497	.619	0.413
H5b	Stress	Prevention behaviors	0.013	0.026	0.023	0.592	.554	0.390
H6	Self-efficacy	Prevention behaviors	0.243	0.335	0.051	4.799	.000	0.390

C.R. = critical ratio, RW = regression weight, SE = standard error, SEM = structural equation modelling, SMC = squared multiple correlation, SRW = standardized regression weight.

## 5. Discussion

This study examined the structural relationships among organizational culture, prevention environment, infection control knowledge, attitudes, job stress, self-efficacy, and prevention behaviors among nurses. The SEM results provided several key insights into how individual and contextual factors interact to shape infection prevention practices.

First, the nonsignificant relationship between organizational culture and infection control knowledge suggests that culture alone may not directly enhance knowledge acquisition. This aligns with findings that organizational culture sets the tone for behavior and norms but may not be sufficient to drive individual learning unless paired with formal education or training programs.^[4,18]^ In contrast, the strong positive association between organizational culture and infection control attitudes supports earlier research demonstrating that supportive cultures foster positive mindsets toward safety practices.^[10,15]^ Leadership commitment, open communication, and shared values likely contribute to these outcomes. Furthermore, the direct positive effect of organizational culture on prevention behaviors reinforces the value of creating environments where infection control is embedded in daily routines and reinforced through institutional expectations.^[3,12]^

The prevention environment showed mixed effects. Although it was not significantly related to attitudes, it had a significant negative association with job stress, consistent with previous findings that adequate resources reduce uncertainty and workload-related strain.^[5,24]^ However, its lack of significant impact on prevention behaviors suggests that infrastructure and protocols alone may not guarantee compliance. Motivation, behavioral skills, and organizational reinforcement likely play mediating roles.^[20]^ This implies that material support must be integrated with training and motivational strategies to yield behavioral change.

Contrary to prior studies, infection control knowledge did not significantly influence attitudes, self-efficacy, or behaviors. This unexpected result challenges traditional models that treat knowledge as a foundational input to behavior.^[7,26,28]^ One possible explanation is that knowledge remains abstract unless grounded in hands-on experience or supported by motivational factors. In fact, the negative, though nonsignificant, direction of the relationship between knowledge and behavior may suggest overconfidence or disengagement among those who possess theoretical knowledge but lack practical skills or organizational support.^[29]^ These findings underscore the need to integrate experiential learning, peer modeling, and contextualized education into infection control training.

Attitudes did not significantly reduce stress, indicating that personal perceptions may be insufficient to buffer the effects of institutional stressors. However, attitudes were strongly associated with self-efficacy, affirming that a positive mindset enhances confidence in performing infection control tasks.^[16,32]^ This relationship suggests that fostering constructive attitudes (through peer support, recognition programs, or leadership engagement) can build self-belief and motivation. The nonsignificant link between attitudes and behavior challenges core assumptions of the IMB model, suggesting that other variables such as habit, supervision, or organizational norms may moderate this pathway.

Job stress did not significantly affect either self-efficacy or prevention behaviors. These results contrast with literature suggesting that stress impairs cognitive function and adherence to safety protocols.^[11,36]^ One plausible interpretation is that in high-stakes healthcare settings, infection control behaviors may be perceived as nonnegotiable professional duties, upheld regardless of psychological strain. Alternatively, workplace resilience, peer norms, or structured routines might buffer the impact of stress on performance.^[12,35]^ While stress reduction initiatives are valuable for overall well-being, they may not directly enhance behavioral compliance with infection protocols.

Finally, the significant positive effect of self-efficacy on prevention behaviors affirms the critical role of confidence in executing infection control practices. This result aligns with both the IMB model and broader behavioral theories, reinforcing that individuals who believe in their capability are more likely to act consistently and correctly.^[37,38]^ Practical interventions to enhance self-efficacy (such as simulation-based workshops, feedback systems, and mentoring) can therefore be highly effective in improving compliance with infection control measures.

In summary, the findings highlight that a supportive organizational culture and enhanced self-efficacy are essential drivers of infection prevention behaviors. While knowledge and infrastructure are necessary, they are not sufficient alone to drive behavioral adherence. The study challenges simplified models of behavior change and underscores the value of multilevel interventions that address attitudes, motivation, institutional support, and psychological empowerment.

## 6. Conclusion

### 6.1. Theoretical contribution

This study contributes to the literature on infection prevention behaviors by addressing gaps in previous research through the application of the IMB model. Unlike earlier studies that explored individual predictors such as knowledge, attitudes, or environmental factors in isolation, this study incorporates these variables within an integrated framework, highlighting their interrelationships and potential directional pathways.^[5,6]^ While organizational culture and environmental support have often been treated as peripheral, this study proposes that they may play a central role in shaping infection prevention behaviors among nurses.

A notable theoretical implication is the role of infection control self-efficacy as a mediator between attitudes and prevention behaviors. While prior studies have emphasized self-efficacy as a determinant of health behavior,^[28,38]^ this study suggests that its influence may be particularly salient in high-stress healthcare settings. Additionally, the finding that job stress did not exhibit a significant direct effect on behavior challenges conventional assumptions and raises the possibility that professional norms or institutional expectations may buffer the adverse effects of stress.^[12,35]^

Finally, this study offers insights into how a supportive prevention environment and positive organizational culture may reinforce motivation and behavioral skills. Prior research often examined physical infrastructure or administrative policies independently; in contrast, this study suggests their combined influence may be more impactful.^[3,22]^ For researchers, this underscores the utility of comprehensive models like IMB in unpacking the multilayered determinants of healthcare behaviors. Future studies should further investigate how specific cultural attributes, leadership styles, or institutional supports interact with psychological resources to shape sustainable infection prevention practices.

### 6.2. Practical implication

This study offers actionable insights for healthcare practitioners, managers, and policymakers aiming to enhance infection prevention in clinical settings. By identifying infection control self-efficacy as a significant driver of prevention behaviors, this research emphasizes the need for targeted training programs. For example, healthcare managers can implement simulation-based workshops to improve nurses’ confidence in applying infection control protocols, such as handling protective equipment or managing isolation practices. Such programs should provide immediate feedback and foster skill acquisition to reinforce self-efficacy.

Healthcare organizations should also prioritize fostering a positive infection control culture. Managers can cultivate this culture by encouraging open communication, recognizing adherence to infection protocols, and ensuring that infection prevention is embedded in daily practices. For example, creating a recognition system for staff who excel in infection prevention can strengthen compliance and morale. Additionally, regular team discussions about infection prevention challenges and successes can build collective accountability and shared responsibility among staff.

Policymakers can use these findings to guide resource allocation. This study highlights the critical role of a well-supported prevention environment, suggesting that policies should ensure the availability of PPE, robust administrative protocols, and up-to-date training resources. For instance, national health agencies could mandate and fund annual infection prevention training programs tailored to specific healthcare settings.

For nurses and frontline healthcare workers, this research underscores the importance of self-awareness regarding their attitudes and self-efficacy. Practitioners can engage in self-reflection or peer mentoring programs to identify barriers to their own adherence to protocols. Ultimately, these interventions aim to build a healthcare environment where infection prevention behaviors are not only a requirement but also a professional norm that practitioners are confident and motivated to uphold.

In summary, this study offers both theoretical and practical contributions to understanding infection prevention behaviors among nurses. Theoretically, it extends the application of the IMB model by integrating organizational and psychological dimensions, such as culture and self-efficacy, into a unified framework. Practically, it identifies actionable levers (such as enhancing self-efficacy through training and fostering a positive organizational culture) that can be targeted by healthcare managers and policymakers to improve infection control compliance. By bridging theoretical insights with field-based implications, the study provides a foundation for more effective and sustainable infection prevention strategies in healthcare settings.

### 6.3. Limitations and future research directions

This study has several limitations that should be acknowledged to contextualize the findings and guide future research. First, the internal consistency of the infection control knowledge scale was relatively low (Kuder–Richardson 20 = .52), limiting the reliability of this measure. Therefore, findings related to knowledge should be interpreted with caution. Second, the cross-sectional design restricts our ability to draw causal inferences, even though SEM allows for the modeling of directional relationships. Longitudinal or experimental studies are recommended to more robustly examine causal pathways. Third, the use of self-reported data may have introduced response bias, as participants could have overstated their infection prevention behaviors due to social desirability. Future research could incorporate observational or multisource data to enhance measurement validity. Fourth, the sample was limited to nurses from 2 mid-sized hospitals in South Korea, which may limit the generalizability of the findings to other healthcare contexts, such as tertiary or rural healthcare settings, or different cultural environments. Lastly, while this study focused on direct and mediated relationships, future research should examine moderating variables, such as leadership style, organizational support, or team dynamics, to provide a more comprehensive understanding of infection prevention behaviors.

## Author contributions

**Conceptualization:** Sun Ok Kim, Moon Suk Sim.

**Data curation:** Sun Ok Kim.

**Formal analysis:** Sun Ok Kim.

**Investigation:** Sun Ok Kim, Moon Suk Sim.

**Methodology:** Sun Ok Kim.

**Validation:** Sun Ok Kim.

**Writing – original draft:** Sun Ok Kim.

**Writing – review & editing:** Sun Ok Kim, Moon Suk Sim.
